# Supplementary material for: Tribological Properties of Nitrate Graphite Foils
Source: Nanomaterials (Basel). 2024 Sep 15;14(18):1499. doi: 10.3390/nano14181499 (PMC11434555; doi:10.3390/nano14181499)
Supplement: Supplementary file 1 [file nanomaterials-14-01499-s001.zip › X-ray data/misorientation/0001/160-200/dens_13/RD/177.pdf]

Raw data

Sample name:		X-ray	: Cu / 40 kV / 30 mA		Counter	:	Scintillation counter
File	: 177.raw	Goniometer	: Ultima IV				
Comment	:	Attachment	: MPA-ML4(with gamma) for stress				
Date	: May-13-24 14:46:55	Filter	: K-beta filter		Scan mode	:	Continuous
Operator	: Administrator	I.Monochro	: CBO		Scan speed	:	5.00 deg./min.
		C.Monochro	: Not installed		Sampling width	:	0.05 no unit
		DivSlit	: Open		Scan axis	:	theta
		DivH.L.Slit	: 10.00mm		Scan range	:	10.00 -> 80.00 no unit
		SctSlit	: 2/3?‹|2/3deg.		Fixed angle	:	0.00 no unit
		RecSlit	: 0.30mm				

Memo :

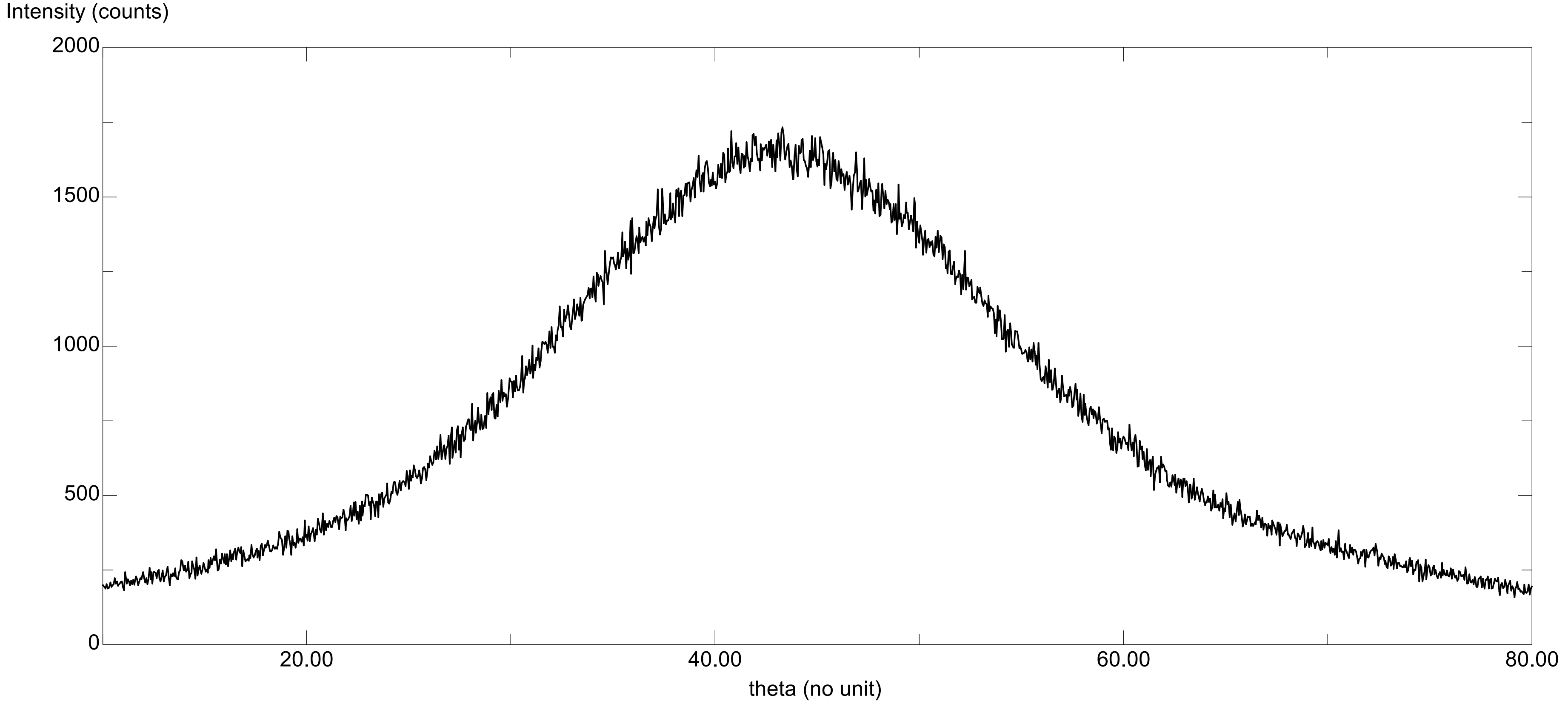

Integral Int. Calculation (History)

Sample name: File : 177.raw   Date : May-13-24 14:46:55   Operator : Administrator
Comment :   Memo :
Peak calc. method: FW2/3M

h	k	l	Start	Stop	Peak	d-value	FWHM	Max. int.	Gross int.	Integ.w	Integ. int.
0	0	0	10.00	no unit80.00	no unit43.35	no unit2.09	25.56	no unit1453 counts	815934.06	28.085	40796.70

[Cut]
[Slit Correction]
[LP Corr.]

[Absorp. Corr.]
[Norm.]
[Smoothing] Method : Averaging   Smoothing Points: 11
[BG subtraction] Method : Averaging at the end
Low angle : 5   High angle : 12
[Ka2 elimination]

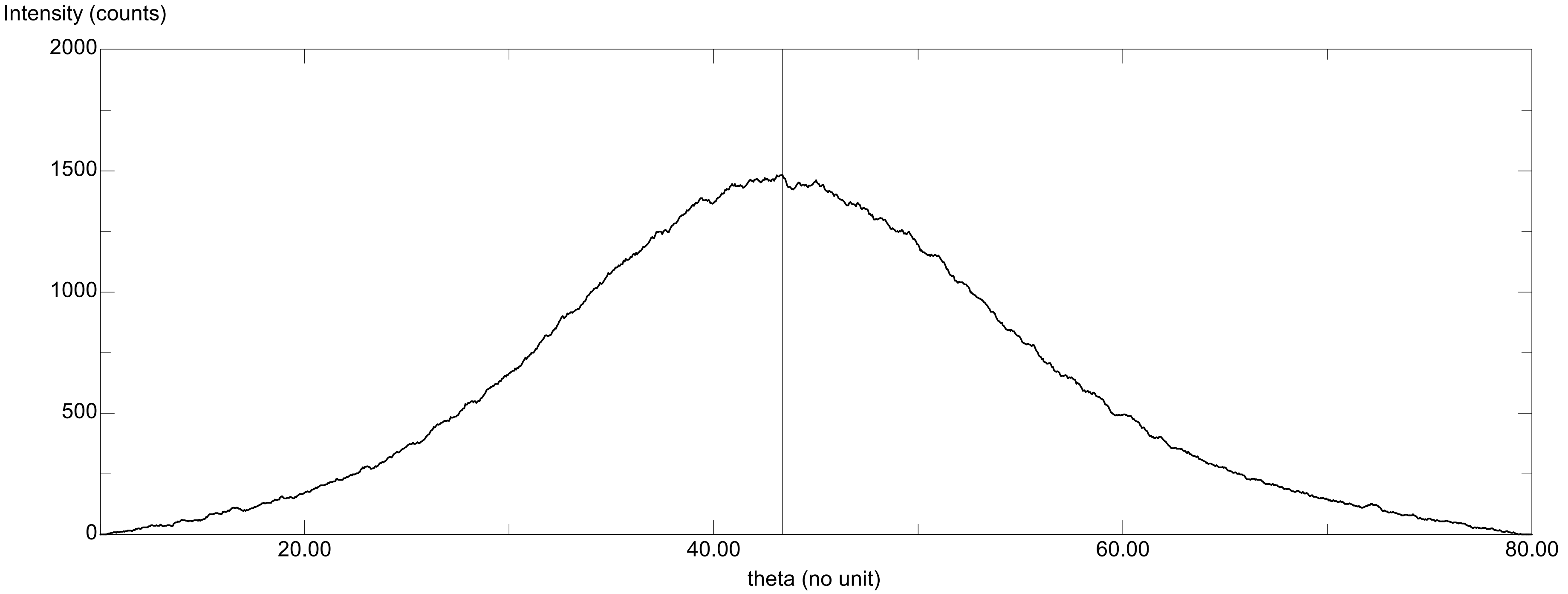

Integral int. sum table

Sample name: File : 177.raw Date : May-13-24 14:46:55 Operator : Administrator
Memo :
Peak calc. method: FW2/3M

No.	h k l	Start	Stop	Peak	d-value	FWHM	Max. int.	Gross int.	Integ.w	Integ. int.	Comment
1	0 0 0	10.00	80.00	43.35	2.09	25.56	1453	815934.06	28.085	40796.70	
